# HOXA10 promotes *Gdf5* expression in articular chondrocytes

**DOI:** 10.1038/s41598-023-50318-7

**Published:** 2023-12-20

**Authors:** Tomohiko Murakami, Lerdluck Ruengsinpinya, Yoshifumi Takahata, Yuri Nakaminami, Kenji Hata, Riko Nishimura

**Affiliations:** 1 Department of Molecular and Cellular Biochemistry, Osaka University Graduate School of Dentistry, 1-8 Yamada-Oka, Suita, Osaka 565-0871 Japan; 2 Department of Oral Surgery and Oral Medicine, Faculty of Dentistry, Srinakharinwirot University, Bangkok, 10110 Thailand

**Keywords:** Gene expression, Gene regulation, Reporter genes, Osteoarthritis

## Abstract

Growth differentiation factor 5 (GDF5), a BMP family member, is highly expressed in the surface layer of articular cartilage. The *GDF5* gene is a key risk locus for osteoarthritis and *Gdf5*-deficient mice show abnormal joint development, indicating that GDF5 is essential in joint development and homeostasis. In this study, we aimed to identify transcription factors involved in *Gdf5* expression by performing two-step screening. We first performed microarray analyses to find transcription factors specifically and highly expressed in the superficial zone (SFZ) cells of articular cartilage, and isolated 11 transcription factors highly expressed in SFZ cells but not in costal chondrocytes. To further proceed with the identification, we generated *Gdf5-*HiBiT knock-in (*Gdf5-*HiBiT KI) mice, by which we can easily and reproducibly monitor *Gdf5* expression, using CRISPR/Cas9 genome editing. Among the 11 transcription factors, *Hoxa10* clearly upregulated HiBiT activity in the SFZ cells isolated from *Gdf5*-HiBiT KI mice. *Hoxa10* overexpression increased *Gdf5* expression while *Hoxa10* knockdown decreased it in the SFZ cells. Moreover, ChIP and promoter assays proved the direct regulation of *Gdf5* expression by HOXA10. Thus, our results indicate the important role played by HOXA10 in *Gdf5* regulation and the usefulness of *Gdf5*-HiBiT KI mice for monitoring *Gdf5* expression.

## Introduction

Articular cartilage is a specialized connective tissue that protects epiphyses and enables the smooth movement of joints. Disorders of articular cartilage cause significant musculoskeletal dysfunction and their healing is difficult due to the limited ability of articular cartilage to repair itself^1,2^. Articular cartilage is composed of articular chondrocytes and extracellular matrix and has no blood vessels, lymphatics, or nerves^3,4^. Thus, articular chondrocytes are responsible for the maintenance of articular cartilage.

Osteoarthritis (OA) is a major joint disease that causes joint pain, inflammation, and stiffness due to the destruction of articular cartilage^5–7^, and results from joint degradation that occurs over time, overuse, and injury. Recent human genetic studies have shown linkage between OA and multiple polymorphisms, including in growth differentiation factor 5 (*GDF5*)^8,9^. GDF5, also known as BMP14 and CDMP1, is a member of the BMP family and the TGF-β superfamily, and is highly expressed in the surface layer of articular cartilage, particularly in the developmental stage^10–12^. Secreted GDF5 binds to BMP/TGF-β receptors such as BMP receptor (BMPR)1B and BMPR2^13^, and subsequently activates the SMAD signaling pathway^14^, which play important roles in skeletal development and formation. A polymorphism in the 5′UTR of *GDF5* (+ 104 T/C) decreases *GDF5* expression and is associated with susceptibility to OA^15^. In addition to the *GDF5* polymorphism (+ 104 T/C), other human *GDF5* polymorphisms have been reported that cause mutations in the GDF5 protein and result in skeletal dysplasia^16–18^.

*Gdf5*-deficient mice showed shortened limb long bones, abnormal joint development, and a reduction in the number of phalanges in the second through fifth digits^12^. Conversely, transgenic mice with the targeted expression of *GDF5* exhibited chondrodysplasia with expanded cartilage and accelerated chondrocyte differentiation to hypertrophy^19^. Additionally, GDF5 is a critical factor for the differentiation of human iPSCs toward cartilaginous tissue working in cooperation with BMP2 and TGF-β^20^. Together with human genetic research, these studies indicate that GDF5 is essential for joint development and homeostasis.

Several studies have reported the importance of GDF5 in OA animal models. Decreased GDF5 levels were found to accelerate OA progression in a murine OA model^21^, whereas intra-articular supplementation of GDF5 prevented OA progression in a rat OA model^22^. These findings suggest that the induction of GDF5 in articular cartilage might be useful as therapy for OA and that the identification of transcription factors that promote *Gdf5* expression in articular cartilage would be helpful to develop this. Interestingly, SOX11 and PITX1 have been reported to be involved in the transcriptional regulation of *Gdf5*^23–25^. However, to date, no technology has been established to promote *GDF5* expression in articular cartilage.

*Gdf5* is highly expressed in the superficial zone of articular cartilage, indicating that transcription factors that regulate its expression are predominantly expressed in the superficial zone (SFZ) cells. In this study, we attempted to identify transcription factors that induce *Gdf5* expression in SFZ cells. To achieve this, we established a two-step screening approach combining microarray analyses with a newly developed *Gdf5*-monitoring system based on CRISPR/Cas9 genome editing. The screening and biochemical analyses indicated that HOXA10 promotes *Gdf5* expression through direct binding to the gene promoter. We believe that our findings and the *Gdf5*-monitoring system can contribute to developing a novel therapy for OA.

## Results

### Transcription factors predominantly expressed in superficial zone of articular chondrocytes

**Figure 1:**
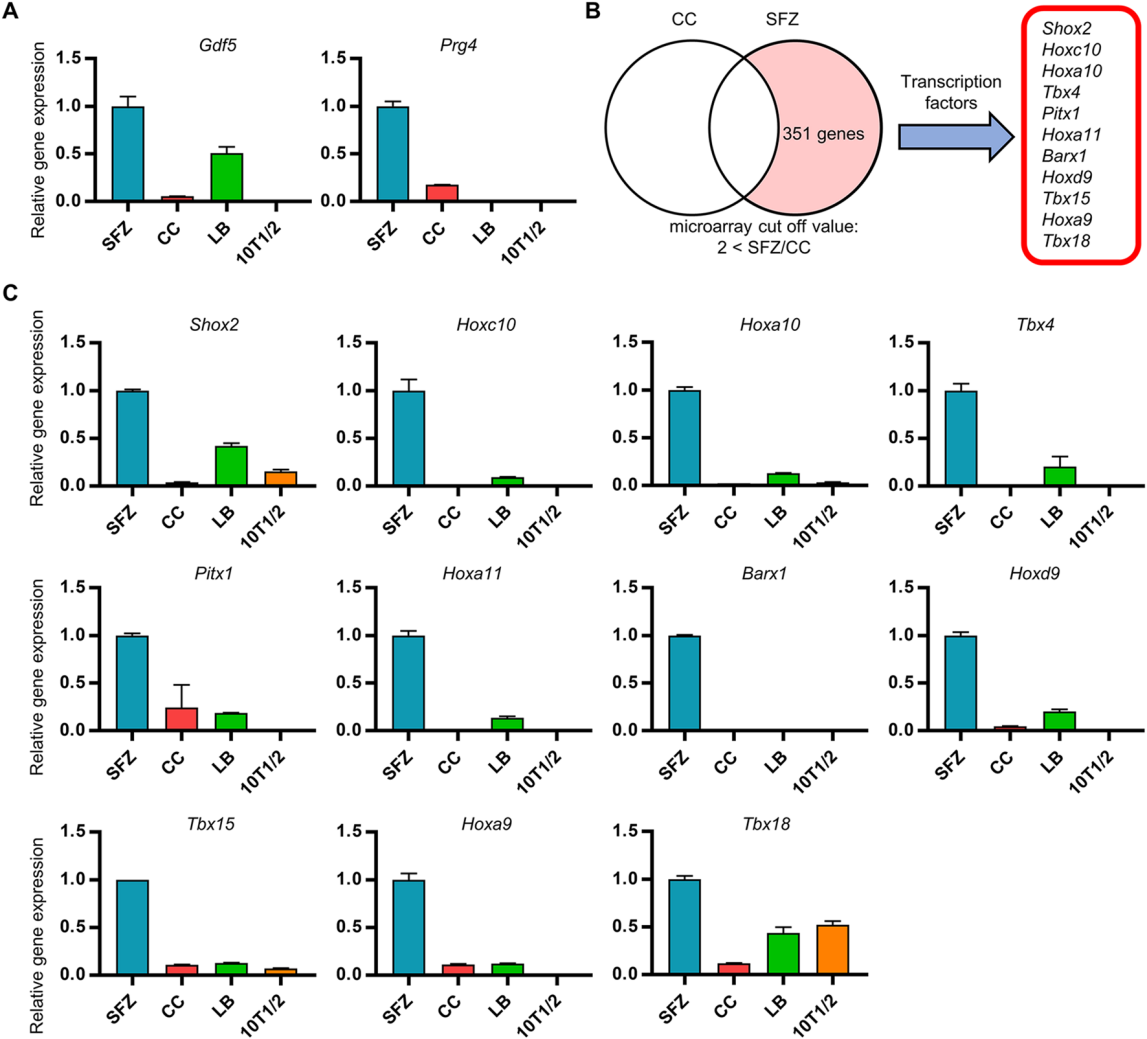
Identification of transcription factors predominantly expressed in articular cartilage cells. (**A**) Total RNA was isolated from SFZ, CC, LB, and C3H10T1/2 (10T1/2) cells. *Gdf5* and *Prg4* expression was analyzed by RT-qPCR in triplicate. (**B**) Total RNA from SFZ cells and CC was analyzed by microarray. Genes with expression exceeding 100 in terms of the SFZ raw values were counted as genes expressed in SFZ cells. The number indicates the number of genes expressed more than two-fold in SFZ cells compared with the level in CC. The panel on the right shows 11 transcription factors among the genes with expression in SFZ cells more than two-fold that in CC. (**C**) Total RNA was isolated from SFZ, CC, LB, and 10T1/2 cells. Indicated gene expression was analyzed by RT-qPCR in triplicate. Data are the mean ± SEM.

To identify the transcription factors involved in *Gdf5* regulation of SFZ cells, we first compared the expression of *Gdf5* and *Prg4*, both of which are specific gene markers of SFZ cells^26,27^, in SFZ cells, costal chondrocytes (CC), and chondrogenic cells, including limb bud (LB) cells and C3H10T1/2 cells. As expected, SFZ cells expressed high levels of *Gdf5* and *Prg4*, whereas CC and C3H10T1/2 cells expressed very little of them (Fig. 1A). Interestingly, LB cells expressed a moderate level of *Gdf5* but no *Prg4* (Fig. 1A), indicating that LB tissues contain precursor cells of SFZ cells. Because *Gdf5* is an earlier-stage gene marker of SFZ cells than *Prg4*^11^, this is very reasonable. These results suggest the utility of comparing these cells to identify transcription factors predominantly and specifically expressed in SFZ cells.

To search for SFZ-specific transcription factors, we performed microarray analyses using SFZ and CC, and found that 11 transcription factors were highly and predominantly expressed in SFZ cells (Fig. 1B and Table 1). Unexpectedly, the gene expression level of *Sox11* in SFZ cells was comparable to that in CC (Table 1). The expression levels of the 11 genes were confirmed by RT-qPCR analyses using total RNA from SFZ, CC, LB, and C3H10T1/2 cells (Fig. 1C). These results suggest that the 11 transcription factors are specific to SFZ cells, and might play roles in the development and/or homeostasis of articular cartilage.

**Table 1:** List of transcription factor genes predominantly expressed in articular chondrocytes by microarray analysis.

Probe Set ID	[SFZ] (raw)	[CC] (raw)	SFZ/CC (fold)	Gene symbol
1420559_a_at	280.5	1.2	232.5	*Shox2*
1439798_at	223.7	4.0	55.6	*Hoxc10*
1431475_a_at	847.9	18.9	44.9	*Hoxa10*
1456033_at	194.5	6.9	28.0	*Tbx4*
1419514_at	348.2	17.3	20.1	*Pitx1*
1420414_at	339.0	23.4	14.5	*Hoxa11*
1423342_at	287.0	27.3	10.5	*Barx1*
1419126_at	204.2	25.4	8.1	*Hoxd9*
1422195_s_at	659.4	96.1	6.9	*Tbx15*
1455626_at	600.8	133.5	4.5	*Hoxa9*
1429974_at	219.9	108.1	2.0	*Tbx18*
1436790_a_at	618.9	774.4	0.8	*Sox11*

Eleven transcription factor genes with more than two-fold increase in SFZ/CC ratio and *Sox11* are shown. *SFZ* superficial zone cells, *CC* costal chondrocytes.

### Development of *Gdf5* expression monitoring system

**Figure 2:**
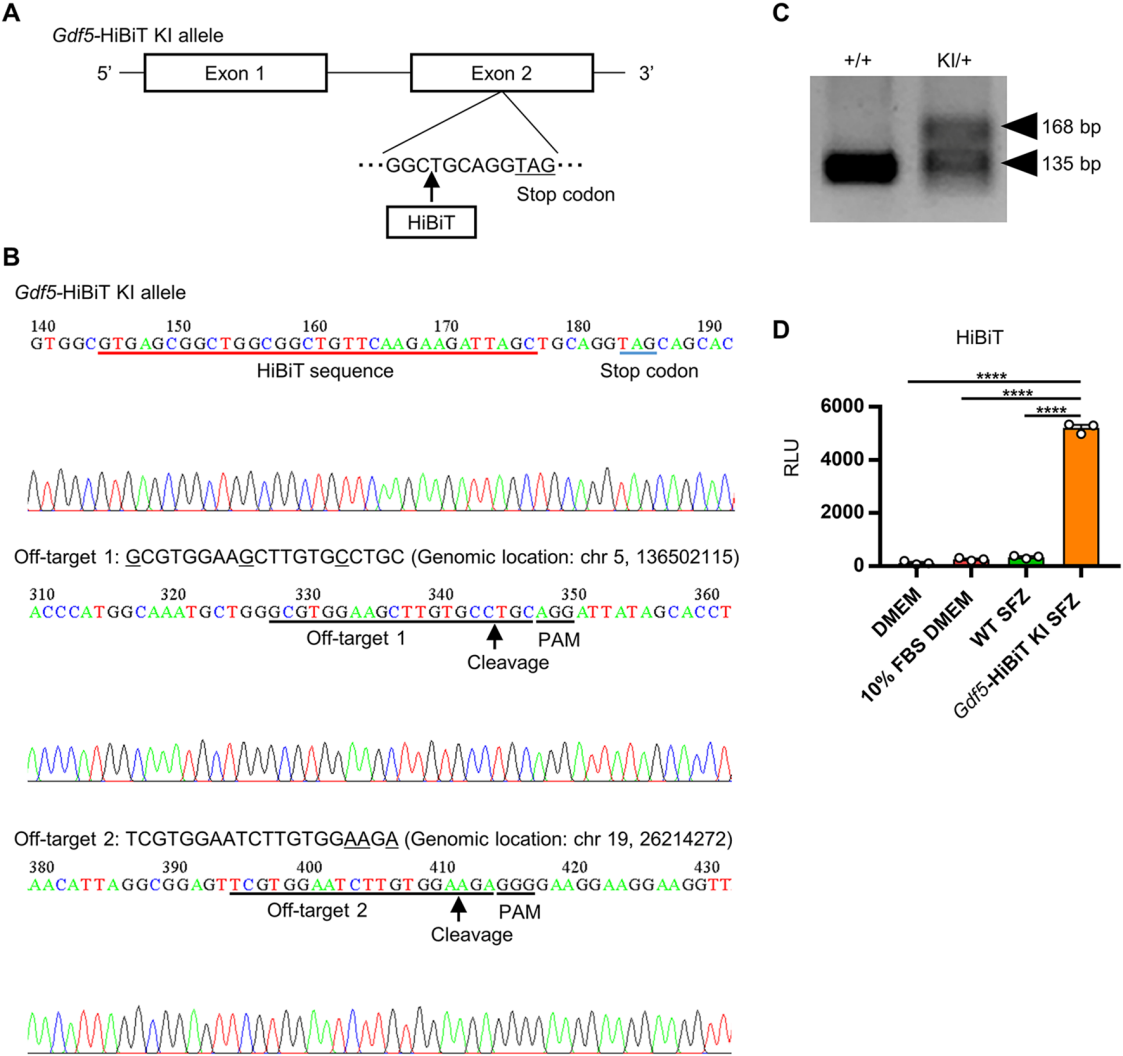
Generation of *Gdf5*-HiBiT screening system. (**A**) Schematic diagram of *Gdf5-*HiBiT allele. The stop codon of *Gdf5* and the knocked-in position of HiBiT tag are indicated. (**B**) *Gdf5-*HiBiT KI mice were generated using the Technique for Animal Knockout system by Electroporation (TAKE) method based on CRISPR/Cas9. Genomic DNA sequence analysis of the *Gdf5* gene was performed, which confirmed that the genome of *Gdf5*-HiBiT KI allele had been edited correctly. Two possible off-target sites for the CRISPR gRNA (target sequence: TCGTGGAATCTTGTGGCTGC) are shown. Genomic DNA sequence analysis of two off-target sites was performed, which confirmed that the sequences around off-target sites were intact. (**C**) Genomic PCR analysis of WT and *Gdf5-*HiBiT KI mice was performed. A representative result is shown. The original gel is presented in Supplementary Fig. 1. (**D**) SFZ cells were isolated from WT and *Gdf5-*HiBiT KI mice. The cells were cultured for 2 days and then the supernatants were collected. DMEM, 10% FBS DMEM, and supernatants of WT and *Gdf5-*HiBiT KI SFZ cells were subjected to HiBiT measurement (n = 3). RLU: relative light unit. Data are the mean ± SEM (****: *P* < 0.0001).

We planned to develop a system for monitoring *Gdf5* expression that allows us to perform high-throughput assays as well as identify the transcription factors that induce *Gdf5* expression. HiBiT® is a small peptide of only 11 amino acids and is a highly sensitive tag-protein that can be detected by the antibody-free NanoLuc luciferase system^28^. Because the HiBiT-tag sequence is very short, HiBiT knock-in (KI) cells or mice with expression equivalent to the endogenous level of candidate genes can be generated using CRISPR/Cas9 gene editing. We therefore generated *Gdf5*-HiBiT KI mice by CRISPR/Cas9 genome editing (Fig. 2A–C). Genomic DNA sequence analysis of the *Gdf5* gene and two possible off-target sites confirmed that the genome of *Gdf5*-HiBiT KI mice had been edited correctly. *Gdf5-*HiBiT KI mice were viable, fertile, and exhibited no gross abnormalities. We isolated SFZ cells from articular cartilage of *Gdf5-*HiBiT KI mice and detected clear and reproducible *Gdf5*-HiBiT signals of the supernatants of the SFZ cells isolated from these mice (Fig. 2D). Thus, we succeeded in developing a *Gdf5* expression monitoring system by which we can perform high-throughput assays using primary isolated cells such as SFZ cells.

### HOXA10 promotes *Gdf5* expression in articular chondrocytes

**Figure 3:**
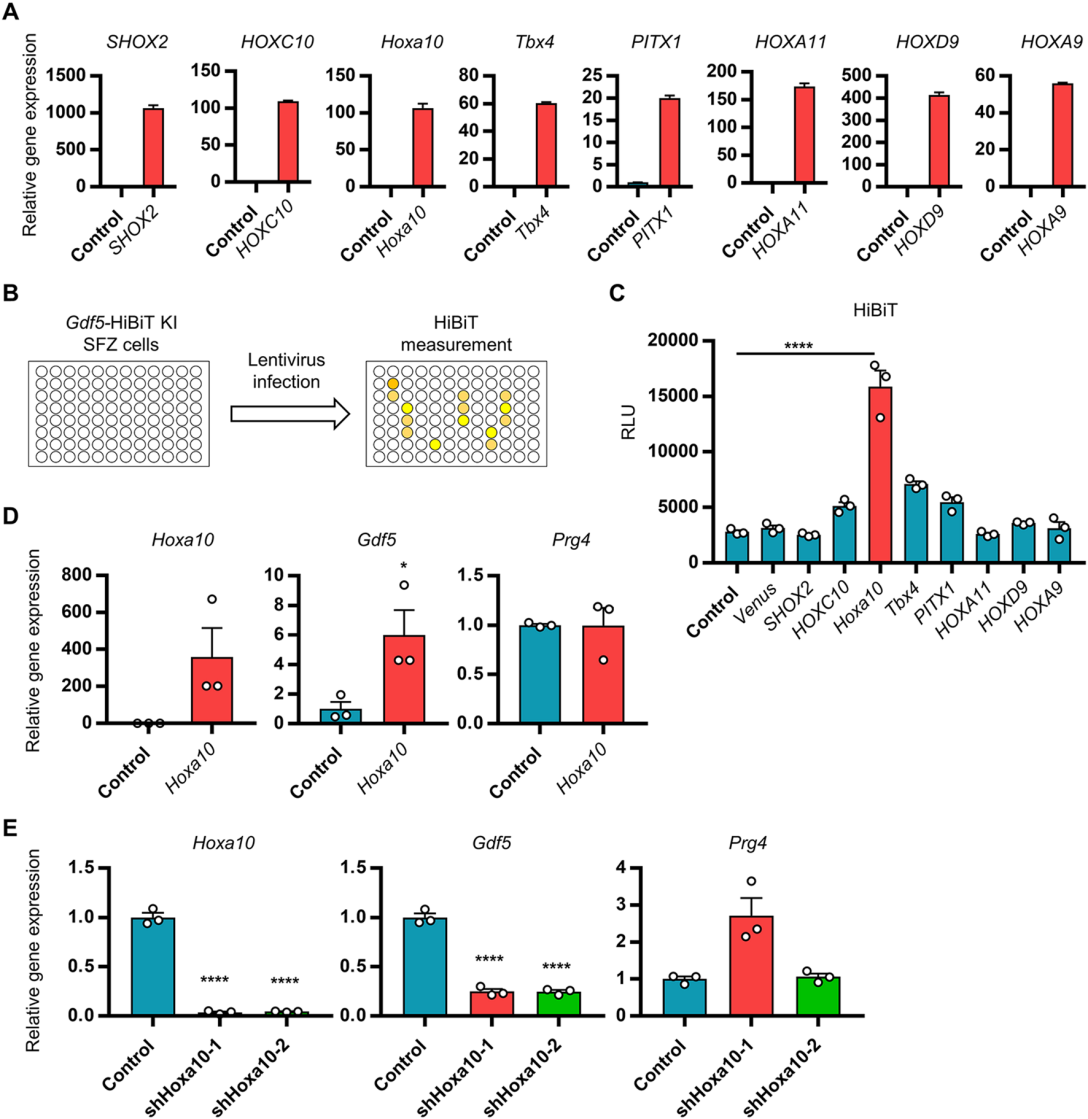
Role of HOXA10 in *Gdf5* expression in articular cartilage cells. (**A**) SFZ cells isolated from WT mice were infected with empty (control) or indicated lentiviruses. Indicated gene expression was analyzed by RT-qPCR in duplicate. (**B**) Schematic diagram of *Gdf5*-HiBiT screening system. (**C**) SFZ cells were isolated from *Gdf5-*HiBiT KI mice and were plated on a 96-well plate. *Gdf5-*HiBiT KI SFZ cells were infected with the indicated lentiviruses. One day later, the medium was changed. The cells were cultured for 2 days and then the supernatants were collected. The supernatants were subjected to HiBiT measurement (n = 3). RLU: relative light unit. (**D**) SFZ cells isolated from WT mice were infected with empty (control) or FLAG-*Hoxa10* lentiviruses. *Hoxa10*, *Gdf5*, and *Prg4* expression was analyzed by RT-qPCR (n = 3). (**E**) SFZ cells isolated from WT mice were infected with empty (control), shHoxa10-1, or shHoxa10-2 lentiviruses. *Hoxa10*, *Gdf5*, and *Prg4* expression was analyzed by RT-qPCR (n = 3). Data are the mean ± SEM (*: *P* < 0.05, ****: *P* < 0.0001).

We aimed to identify the transcription factors that promote *Gdf5* expression in SFZ cells using *Gdf5*-HiBiT mice, from among the transcription factors predominantly expressed in SFZ cells (Fig. 1B,C). To achieve this, we first generated lentiviruses expressing these transcription factors. However, unfortunately, *Barx1*, *Tbx15*, and *Tbx18* lentiviruses did not express their exogenous proteins in SFZ cells, although we do not know the reason for this. Therefore, we focused on *SHOX2*, *HOXC10*, *Hoxa10*, *Tbx4*, *PITX1*, *HOXA11*, *HOXD9*, and *HOXA9* lentiviruses (Fig. 3A). To determine whether these transcription factors induce *Gdf5* expression, SFZ cells isolated from *Gdf5-*HiBiT KI mice were plated in 96-well plates and the transcription factors were introduced by a lentiviral system (Fig. 3B). HiBiT activity of the supernatants from each well was determined using the HiBiT assay system. We found that *Hoxa10* overexpression clearly increased HiBiT activity (Fig. 3C). To confirm that *Hoxa10* is involved in *Gdf5* expression, we examined the effect of *Hoxa10* overexpression in SFZ cells by performing RT-qPCR analysis. Consistent with the HiBiT assay results, *Hoxa10* overexpression promoted *Gdf5* expression but not *Prg4* expression (Fig. 3D). Conversely, *Hoxa10* knockdown suppressed *Gdf5* expression in SFZ cells, but not *Prg4* expression (Fig. 3E). These results indicate that HOXA10 is a transcription factor that promotes *Gdf5* expression in SFZ cells and that our assay system using *Gdf5*-HiBiT KI mice is very useful.

### HOXA10 promotes *Gdf5* expression in LB cells

**Figure 4:**
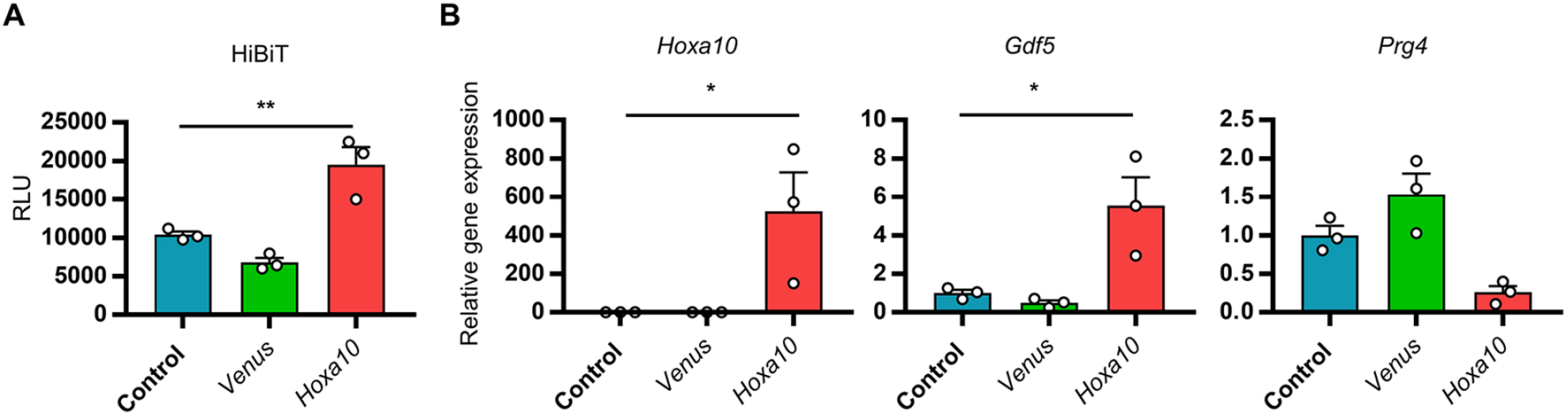
Role of HOXA10 in *Gdf5* expression in LB cells. (**A**) LB cells were isolated from *Gdf5-*HiBiT KI mice and plated on a 96-well plate. The cells were infected with empty (control), *Venus*, or FLAG-*Hoxa10* lentiviruses. One day after infection, the medium was changed. The cells were cultured for 2 days and then the supernatants were collected. The supernatants were subjected to HiBiT measurement (n = 3). RLU: relative light unit. (**B**) LB cells isolated from WT mice were infected with empty (control), *Venus*, or FLAG-*Hoxa10* lentiviruses. *Hoxa10*, *Gdf5*, and *Prg4* expression was analyzed by RT-qPCR (n = 3). Data are the mean ± SEM (*: *P* < 0.05, **: *P* < 0.01).

*Gdf5* is highly expressed in the developing limb at the site where the joint cavity is formed^11,12^. Additionally, *Hoxa10* was found to be moderately expressed in LB cells (Fig. 1C). We were therefore curious to find out whether HOXA10 stimulates the differentiation of LB cells to *Gdf5*-positive SFZ-like cells. *Hoxa10* overexpression increased *Gdf5*-HiBiT in *Gdf5-*HiBiT KI LB cells (Fig. 4A). Consistent with this, *Hoxa10* overexpression increased *Gdf5* expression in LB cells (Fig. 4B). However, *Gdf5* overexpression failed to promote *Prg4* expression (Fig. 4B) as in SFZ cells (Fig. 3D). Taken together, these results indicate that HOXA10 plays a role in the development of articular cartilage from limbs, but is not involved in the regulation of *Prg4* expression. *Prg4* would be regulated by other transcription factors during the development of articular cartilage.

### HOXA10 binds to and activates the *Gdf5* gene promoter

**Figure 5:**
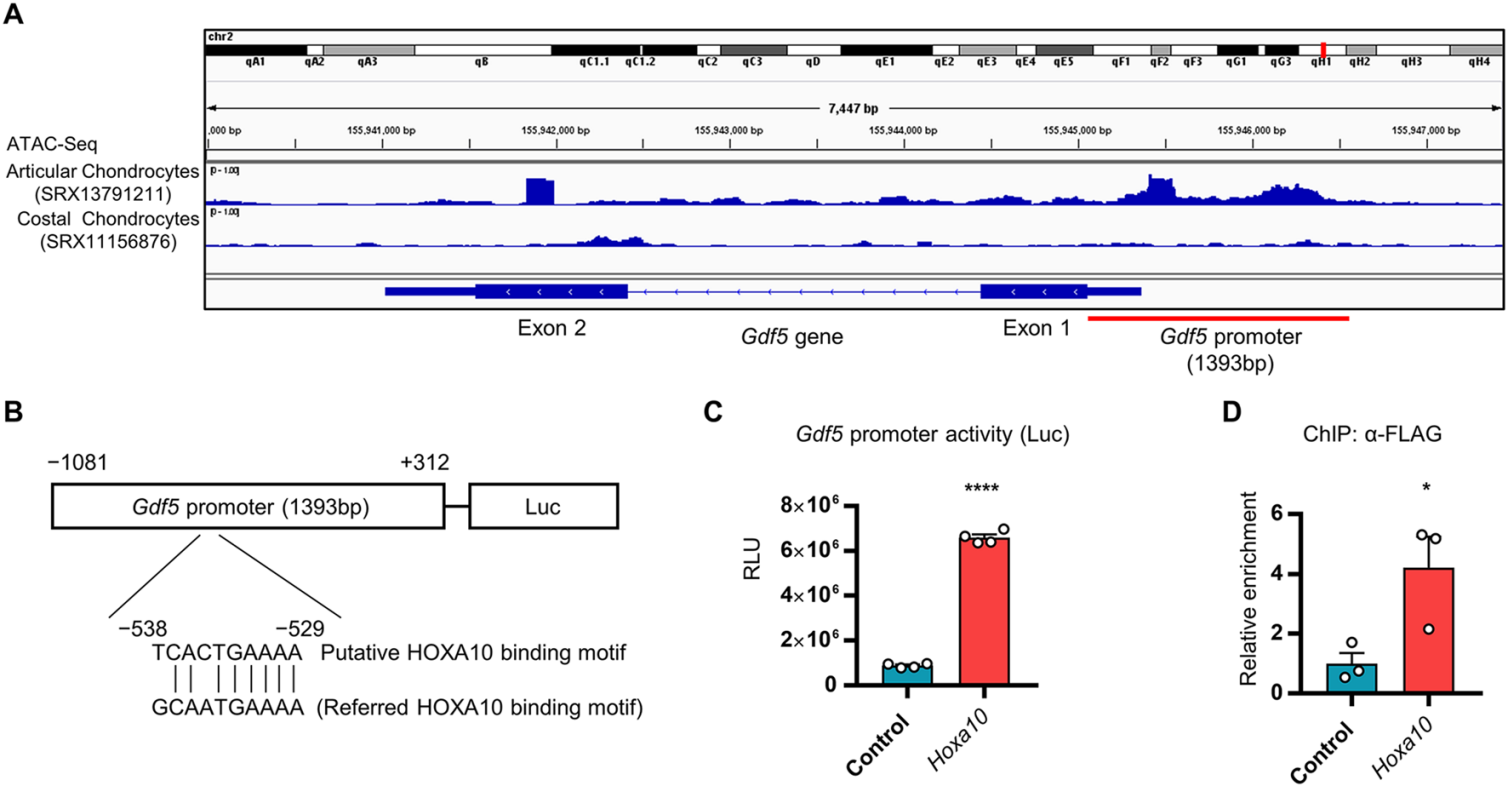
Effect of HOXA10 on the *Gdf5* gene promoter. (**A**) The ATAC-Seq database (articular chondrocytes: SRX13791211, costal chondrocytes: SRX11156876) from ChIP-Atlas was analyzed by integrative genomics viewer IGV2.8.6. A *Gdf5* gene promoter (1393 bp) exhibits an open chromatin region on the *Gdf5* gene in articular chondrocytes. (**B**) Schematic diagram of luciferase reporter construction with mouse *Gdf5* gene promoter (− 1081 to + 312). A putative HOXA10 binding motif (− 538 to − 529) is shown with reference to previous study^30^. (**C**) HEK293T cells were transfected with empty or FLAG-*Hoxa10* plasmids as well as luciferase reporter plasmids with mouse *Gdf5* gene promoter. Cell lysates were subjected to luciferase measurement (n = 4). RLU: relative light unit. (**D**) SFZ cells isolated from WT mice were infected with empty (control) or FLAG-*Hoxa10* lentiviruses. *Gdf5* gene promoter fragments collected by ChIP using anti-FLAG antibody were analyzed by real-time qPCR (n = 3). Data are the mean ± SEM (*: *P* < 0.05, ****: *P* < 0.0001).

To understand the molecular mechanisms by which HOXA10 promotes *Gdf5* expression, we examined the effect of HOXA10 on the *Gdf5* gene promoter. Using a comprehensive epigenetic database, including chromatin immunoprecipitation sequencing (ChIP-seq) analyses and Transposase-Accessible Chromatin sequencing (ATAC-seq), namely, ChIP-Atlas^29^ (https://chip-atlas.org), we analyzed the *Gdf5* gene promoter region. ATAC-seq revealed an open chromatin region around the *Gdf5* gene in articular chondrocytes, in contrast to the case for costal chondrocytes (Fig. 5A). The open chromatin region contained a putative HOXA10 binding motif similar to previous study^30^ (Fig. 5B). Thus, we cloned the open chromatin region as a *Gdf5* gene promoter and performed a promoter assay (Fig. 5B). *Hoxa10* overexpression increased the *Gdf5* promoter activity in HEK293T cells (Fig. 5C). Furthermore, ChIP assay showed that HOXA10 bound to the *Gdf5* gene promoter in SFZ cells (Fig. 5D). These results indicate that HOXA10 binds to the *Gdf5* promoter and activates the transcription of *Gdf5* in SFZ cells.

### HOXA10 is expressed in articular cartilage together with GDF5

**Figure 6:**
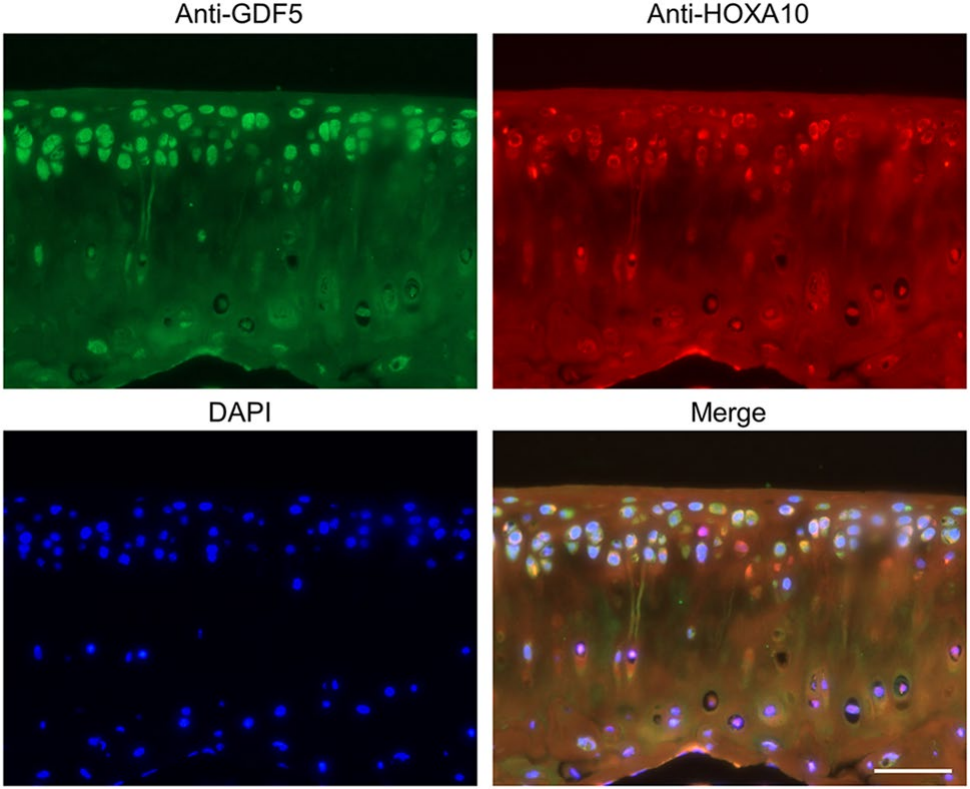
Immunofluorescent analysis of HOXA10 and GDF5 in articular cartilage. Tibial sections from 3-month-old mice were subjected to co-immunostaining with anti-HOXA10 and anti-GDF5 antibodies. DAPI indicates nucleus. Scale bar, 50 μm.

To examine the involvement of HOXA10 in *Gdf5* expression in vivo, we performed immunofluorescent analysis of HOXA10 and GDF5 in articular cartilage. We found that HOXA10 was expressed in superficial zone of articular cartilage as well as GDF5 (Fig. 6). These results suggest that HOXA10 is involved in *Gdf5* expression in articular cartilage and support our finding that HOXA10 promotes *Gdf5* expression.

## Discussion

In this study, we attempted to identify the transcription factors involved in the regulation of *Gdf5* expression, and found that HOXA10 promotes *Gdf5* expression through direct binding to its gene promoter. *Hoxa10* is a member of the abdominal B subclass of homeobox genes and plays key roles in modulating tissue morphogenesis, including that of bone and joint tissues^31,32^. *Hoxa10*-knockout mice are viable but display abnormal reproductive tissues, vertebrae, and spinal nerves with femoral malformations and knee joint degeneration^33,34^. Consistent with these results, our findings indicate that *Hoxa10* contributes to the development of articular cartilage at least partially through *Gdf5* regulation.

Previous studies have been reported that SOX11 and PITX1 are involved in the regulation of *Gdf5* expression^23–25^. In this study, we found that *Pitx1* was highly expressed in articular chondrocytes, but not *Sox11*. However, *Pitx1* overexpression failed to upregulate *Gdf5* expression in articular chondrocytes unlike that in chondrocytes from humanized mice carrying human *GDF5* regulatory elements^25^. The reason is unclear, but one possibility is due to differences in the regulatory elements of human *GDF5* and mouse *Gdf5*. SOX11 has also been reported to increase *HOXA10* expression *in vitro*^35^. Therefore, SOX11 may be involved in the regulation of *Gdf5* expression by promoting *Hoxa10* expression.

Homeobox genes are important in body plan patterning^36^. Therefore, the dysregulation of homeobox genes may be involved in cartilage disorders and lead to the development of OA. Indeed, human *HOXA10* and *HOXA13* tended to be expressed at lower levels in OA chondrocytes, whereas *HOXC8* and *HOXD10* levels were significantly increased^37^. These results and our findings suggest that decreased *HOXA10* in OA chondrocytes leads to decreased *GDF5* expression and consequently OA pathogenesis. *HOXA10* upregulation may contribute to OA treatment through *GDF5* induction.

Although *Hoxa10* plays a role in the development of articular cartilage, *Hoxa10* is not involved in the regulation of *Prg4*, another specific marker gene of SFZ cells. Other mechanisms are thus likely to be involved in this event. This is reasonable because the onset of expression differs between *Gdf5* and *Prg4* during the development of articular cartilage^11^. Thus, identification of the transcription factors that induce *Prg4* expression is also an important part of the development of direct programming of articular cartilage.

With the aging of the population globally, the number of OA patients has increased to an estimated 300 million or more^38,39^. OA is treated by symptomatic therapy such as hyaluronic acid injections into the joint cavity and the administration of pain relief^5–7^. For the treatment of severe OA, artificial joint replacement is considered. Meanwhile, for rheumatoid arthritis (RA), another bone and cartilage disease, several drugs have been developed, such as anti-TNF antibodies and anti-IL-6R neutralizing antibodies^40^, which greatly contribute to preventing RA progression. However, no drug has been developed to prevent the progression of OA. Therefore, therapeutic agents for OA need to be developed. As mentioned above, GDF5 upregulation is a promising drug target for OA. We developed a *Gdf5*-monitoring system that can easily and quantitatively detect *Gdf5* expression with high sensitivity. We successfully used this system to identify HOXA10, indicating that this system is useful for the detection of *Gdf5* expression. Our newly developed HiBiT KI mouse system, in which HiBiT tag is incorporated into the genes of interest in the mouse genome, would provide us with more accurate biological results than HiBiT KI cell lines because we are able to isolate the primary cells of interest from the mice, including SFZ cells, and culture them in 384-well plates as well as 96-well plates. Using this system, we are planning to perform high-throughput screening for chemical compounds that induce *Gdf5* expression, which could lead to the development of OA treatments.

In conclusion, we identified HOXA10 as a transcription factor for *Gdf5* expression via a screening approach combining microarray analysis and a *Gdf5*-monitoring system. HOXA10 promoted *Gdf5* expression in articular cartilage and LB cells, but not *Prg4* expression. *Gdf5* induction, including *Hoxa10* upregulation, would be a therapeutic target for OA and a *Gdf5*-monitoring system can be applied to high-throughput screening to search for *Gdf5* inducers. Thus, our findings provide insights into the regulation of *Gdf5*, methods for monitoring *Gdf5* expression, and therapeutic targets for OA.

## Methods

### Mice

C57BL/6J and ICR mice were obtained from Japan SLC (Shizuoka, Japan). *Gdf5-*HiBiT KI mice were generated using the Technique for Animal Knockout system by Electroporation (TAKE) method based on the CRISPR/Cas9 system, as we previously described^41,42^. A CRISPR gRNA (target sequence: 5′-TCGTGGAATCTTGTGGCTGC-3′) targeting upstream of the *Gdf5* stop codon was complexed with Cas9 protein and introduced together with single-stranded oligodeoxynucleotides (ssODN: 5′-TAAACAGTACGAGGACATGGTCGTGGAATCTTGTGGCGTGAGCGGCTGGCGGCTGTTCAAGAAGATTAGCTGCAGGTAGCAGCACTGGCCCACCTGTCTT-3′) into C57BL6/J pre-nuclear-stage embryos. Genomic DNA sequence analyses of the *Gdf5* gene were performed, which confirmed that the genome of *Gdf5*-HiBiT KI mice had been edited correctly. *Gdf5*-HiBiT KI mice were backcrossed with the C57BL/6J background and were maintained in heterozygous form. To genotype the *Gdf5*-HiBiT KI allele, we used the following primer set: forward 5′-CTTCATCGACTCTGCCAACA-3′ and reverse 5′-ACCTGTGGAGGGGGTAGTCT-3′. Off-target sites of the CRISPR gRNA were searched using Invitrogen TrueDesign Genomic Editor (Thermo Fisher Scientific, Waltham, MA, USA). To analyze genomic DNA sequence of the off-target sites, genomic PCR was performed using the following primer sets: off-target 1 (forward 5′- AGCCCCAGGAACATTTAAGG -3′ and reverse 5′- CAGAAGACCTGGAAGGCTTG -3′) or off-target 2 (forward 5′- TATGAATCCCAGGAGGCAAG -3′ and reverse 5′- CAGGTCTTCGGCAAGAGAAG -3′), followed by DNA sequence analysis. All animal experiments were approved by Osaka University Institutional Animal Experiment Committee and performed in accordance with the regulatory guidelines. The study is reported in accordance with ARRIVE guidelines (https://arriveguidelines.org).

### Cell culture

HEK293T and C3H10T1/2 cells (RIKEN, Ibaraki, Japan) were cultured in Dulbecco’s modified Eagle’s medium (DMEM) (Wako Pure Chemical Industries, Osaka, Japan) supplemented with 10% fetal bovine serum (FBS) (Nichirei Biosciences, Tokyo, Japan) and penicillin–streptomycin-glutamine (Wako Pure Chemical Industries). Articular cartilage SFZ cells were isolated as described previously^26,42^. SFZ cells were maintained in DMEM containing 10% FBS and penicillin–streptomycin-glutamine. Costal chondrocytes were isolated from ribs of 4-day-old mice by digestion with collagenase. The chondrocytes were collected from dispersions and cultured in DMEM supplemented with 10% FBS and penicillin–streptomycin-glutamine. LB cells were isolated from LB of E11.5 embryos by digestion with trypsin and collagenase. The LB cells were collected from dispersions and cultured in DMEM supplemented with 10% FBS and penicillin–streptomycin-glutamine.

### Reverse-transcription quantitative polymerase chain reaction (RT-qPCR) and microarray

Total RNA was isolated from cells using Nucleospin RNA Plus (Macherey–Nagel, Düren, Germany). After denaturation of total RNA, complementary DNA was synthesized from the total RNA with ReverTra Ace qPCR RT Master Mix (Toyobo, Osaka, Japan). Real-time PCR was performed using an ABI Step One Plus real-time PCR system (Applied Biosystems, Waltham, MA, USA) with THUNDERBIRD Probe qPCR Mix or SYBR qPCR Mix (Toyobo). The amount of target mRNA was normalized to that of *β-actin* mRNA. Relative mRNA expression levels were calculated by the comparative threshold cycle (Ct) method. Primer sets used are listed in Supplementary Tables S1 and S2.

For microarray analysis, total RNA was isolated from SFZ cells and CC using a NucleoSpin RNA Plus Kit. cRNA was synthesized using a GeneChip 3’IVT Plus Reagent Kit (Thermo Fisher Scientific). Microarray analysis was performed using the Affymetrix Mouse Genome 430 2.0 Array (Affymetrix, Santa Clara, CA, USA), in accordance with the manufacturer’s protocol.

### HiBiT and luciferase reporter assay

Supernatants from *Gdf5*-HiBiT KI cells with or without lentivirus infection were collected. Their HiBiT signals were measured using the Nano Glo HiBiT Lytic Detection System (Promega, Madison, WI, USA), in accordance with the manufacturer’s protocol. HEK293T cells were transfected with luciferase reporter plasmids containing the *Gfd5* gene promoter as well as pLVSIN-FLAG-*Hoxa10* or pLVSIN-CMV-Pur (empty vector) using X-tremeGene 9 DNA transfection reagent (Sigma-Aldrich, Milwaukee, WI, USA). Their luciferase activities were measured using the Luciferase Assay System (Promega), in accordance with the manufacturer’s protocol.

### Lentivirus plasmid and infection

Lentiviral expression vectors for *HOXA9* (NM_152739.4) tagged with FLAG, *Hoxa10* (NM_008263.4) tagged with FLAG, *HOXA11* (NM_005523.6), *HOXC10* (NM_017409.4), *HOXD9* (NM_014213.4), *Barx1* (NM_007526.4) tagged with FLAG, *Tbx15* (NM_009323.3) tagged with FLAG, *Tbx18* (NM_023814.4) tagged with FLAG, *PITX1* (NM_002653.5), *SHOX2* (NM_003030.4) tagged with FLAG, and *Tbx4* (NM_011536.3) tagged with FLAG were cloned to the pLVSIN-CMV-Pur vector (Takara, Tokyo, Japan). For lentiviral protein transduction, Lenti-X 293T cells (Takara) were transfected with each lentivirus plasmid as well as Lentiviral High Titer Packaging Mix (Takara) using X-tremeGENE 9 (Sigma-Aldrich). The empty vector and/or pLVSIN-Venus was used as a negative control. Lentiviral particle preparation and infection were performed in accordance with the manufacturer’s protocol (Takara). For knockdown experiments, lentiviral pLKO.1 plasmids targeting *Hoxa10* (Hoxa10 shRNA-1 target sequence, 5′-ATCCTTCATTCACCTTTGAG-3′ and Hoxa10 shRNA-2 target sequence, 5′-AAGCAAATGCATTCTATCGTT-3′, were generated in accordance with the manufacturer’s protocol (Addgene, Watertown, MA, USA). Lentiviral particle preparation and infection were performed in accordance with the manufacturer’s protocol (Addgene).

### ChIP-Atlas analysis and *Gfd5* promoter cloning

To identify an open chromatin region on the *Gdf5* gene promoter that differs between articular chondrocytes and costal chondrocytes, a search of ChIP-Atlas, an integrative, comprehensive database (https://chip-atlas.org/), was performed. ATAC-Seq data (articular chondrocytes: SRX13791211, costal chondrocytes: SRX11156876) were analyzed using integrative genomics viewer, IGV2.8.6 (https://software.broadinstitute.org/software/igv/home). The *Gfd5* gene promoter region (− 1081 to + 312) was cloned as an open chromatin region in articular chondrocytes.

### ChIP assay

ChIP analysis was performed using the truChIP Chromatin Shearing Kit (Covaris, Woburn, MA, USA). SFZ cells were infected with control (EV) or FLAG-*Hoxa10* lentivirus. Cells were washed with PBS, followed by chromatin fixation with 1% formaldehyde for 10 min. Sonicated chromatin samples were immunoprecipitated with an anti-FLAG DYKDDDDK antibody (1:100, M185; Medical & Biological Laboratories, Tokyo, Japan). DNA was eluted from immunoprecipitated samples using the SimpleChIP Plus Sonication Chromatin IP Kit (Cell Signaling Technology, Danvers, MA, USA). *Gdf5* gene promoter region fragments were analyzed by qPCR using the following primer pair: forward (5′-TCACTGAAAACCTTGCTTGC-3′) and reverse (5′-AAAAATTACCGCTGCCCTTT-3′).

### Immunofluorescence

Tibias isolated from 3-month-old male mice were fixed in 4% paraformaldehyde and then decalcified with 10% EDTA. The decalcified bones were embedded in paraffin and sectioned. Sections were deparaffinized and hydrated through xylene and graded concentrations of alcohol. Antigen retrieval was performed with hyaluronidase. Slides were incubated overnight with primary antibodies against HOXA10 (GTX37412, GeneTex, Irvine, CA, USA; 1:100) and GDF5 (AF853, R&D Systems; 1:100). Slides were then incubated with secondary antibodies against rabbit IgG [Alexa Fluor 568 (A10042, Thermo Fisher Scientific; 1:500)] and goat IgG [Alexa Fluor 488 (A11055, Thermo Fisher Scientific; 1:500)], followed by DAPI staining. Images were acquired with a DFC7000 T digital camera (Leica, Wetzlar, Germany) under a DM4B microscope (Leica).

### Statistical analyses

Statistical analyses were performed using Prism 7 (GraphPad Software, Boston, MA, USA). Comparisons of two groups were performed using unpaired two-tailed Student’s t test. Comparisons of multiple groups were performed using one-way analysis of variance (ANOVA), followed by Bonferroni’s post hoc test. *P* < 0.05 was considered significant. All data are shown as the mean ± SEM.

### Supplementary Information

Supplementary Information.

## Data Availability

Microarray data have been deposited in the Gene Expression Omnibus database under Accession Number GSE242028. Data and materials are available from the corresponding authors upon reasonable request.
