# Supplementary material for: HOXA10 promotes Gdf5 expression in articular chondrocytes
Source: Sci Rep. 2023 Dec 20;13:22778. doi: 10.1038/s41598-023-50318-7 (PMC10733362; doi:10.1038/s41598-023-50318-7)

Supplementary Information

HOXA10 promotes *Gdf5* expression in articular chondrocytes

Tomohiko Murakami, Lerdluck Ruengsinpinya, Yoshifumi Takahata, Yuri Nakaminami, Kenji Hata, Riko Nishimura

Supplementary Table S1. Primer list for qPCR

Shox2-F: 5'-GAGGAATTGAGCCAGCGACTG-3'
Shox2-R: 5'-CGGCTCCTATAAGGACACCTTTG-3'
Shox2-P: 5'-CCAAACCTGTACTCGGGCCTCAGAGAGC-3'
Hoxc10-F: 5'-AGTCCCAATGAGATCAAGACAGAG-3'
Hoxc10-R: 5'-TGGTGTTTTCTGCCTTTATCTCCTC-3'
Hoxc10-P: 5'-CAAGACCGCAGACTCCAGTCCAGACACC-3'
Hoxa10-F: 5'-AACGCTGCCCTTACACGAAG-3'
Hoxa10-R: 5'-GGACGCTACGGCTGATCTCT-3'
Hoxa10-P: 5'-AACTCCTTCTCCAGCTCCAGCGTCTGG-3'
Tbx4-F: 5'-GCCTCTTCTACCACTGCCTGA-3'
Tbx4-R: 5'-GGGGAGAACGGAAATAGTGATCG-3'
Tbx4-P: 5'-TAGAGACAGTGCCCGCCACCTGGACTT-3'
Pitx1-F: 5'-ACCACCACCGCACGACATG-3'
Pitx1-R: 5'-ACCGCGCTCCTTGTCTGG-3'
Pitx1-P: 5'-AGAACTCCGCCAGCGAATCGTCCGAC-3'
Hoxa11-F: 5'-CAAGGCCGGTGGCTCCG-3'
Hoxa11-R: 5'-GGACAGTTGCAGACGCTTCTC-3'
Hoxa11-P: 5'-TGAAGAAGAACTCTCGCTCCAGCTCTCGGA-3'
Barx1-F: 5'-ATCGAAGAGATCCTCACTGAGCC-3'
Barx1-R: 5'-AGCCTTCAGCACCGCCAG-3'
Barx1-P: 5'-CGCCTGTACGCCGAACTTGAGCAGCT-3'
Hoxd9-F: 5'-CGCCGCAGCAGCAACTTG-3'
Hoxd9-R: 5'-CCGGGTGAGGTACATGTTGAAG-3'
Hoxd9-P: 5'-CGCTGTCCCTACACCAAATACCAGACGCT-3'
Tbx15-F: 5'-GGACTTGAAGCCATCATGGAGA-3'
Tbx15-R: 5'-CTGTACTGCCCCCTTGCTG-3'
Tbx15-P: 5'-CATTCTGGAGACCTCCTGTTCGCACACTG-3'
Hoxa9-F: 5'-GGCGCCTTCTCCGAAAACAA-3'
Hoxa9-R: 5'-GCAGCCGGGTTATTGGGATC-3'
Hoxa9-P: 5'-CTTGTCTCCGCCGCTCTCATTCTCGG-3'
Tbx18-F: 5'-GACAGGAATCCATTTGCCAAAGG-3'
Tbx18-R: 5'-AGGTGAGAGTCCGTAGTGATGG-3'
Tbx18-P: 5'-CTCCAGAATGCGTATGACTCCACCAGAGC-3'
Gdf5-F: 5'-CACCATCACCAGCTTTATTGACAA-3'
Gdf5-R: 5'-CCATCCTTCTCCAAGGCACTG-3'
Gdf5-P: 5'-AACACGTACCTCTGCTTCCTGACCGCA-3'
Prg4-F: 5'-ATCCCATGCTTTCAGATGAGACC-3'
Prg4-R: 5'-CAGCATCCAGAAATAATGACCTCG-3'
Prg4-P: 5'-CGCAGCGTAGTCAGTCCATCCACTGG-3'
β-actin-F: 5'-TTAATTTCTGAATGGCCCAGGTCT-3'
β-actin-R: 5'-ATTGGTCTCAAGTCAGTGTACAGG-3'
β-actin-P: 5'-CCTGGCTGCCTCAACACCTCAACCC-3'

Supplementary Table S2. Human and mouse common primer list for qPCR

hm*Shox2*-F: 5'-CCCTTGTCCTTTCAGGTTCA-3'
hm*Shox2*-R: 5'-ATGCTGGAGTTCTTGCTGGT-3'
hm*Hoxc10*-F: 5'-CGCCTGGAGATTAGCAAGAC-3'
hm*Hoxc10*-R: 5'-GATCCGATTCTCTCGGTTCA-3'
hm*Pitx1*-F: 5'-GGCCTCAACAACATCAACAA-3'
hm*Pitx1*-R: 5'-CGAGTGCTGTTTGGACTTGA-3'
hm*Hoxa11*-F: 5'-GATTTCTCCAGCCTCCCTTC-3'
hm*Hoxa11*-R: 5'-AGTAGCAGTGGGCCAGATTG-3'
hm*Hoxd9*-F: 5'-TGAAGGAGGAGGAGAAGCAG-3'
hm*Hoxd9*-R: 5'-AGCGTCTGGTATTTGGTG-3'
hm*Hoxa9*-F: 5'-CCACGCTTGACACTCACACT-3'
hm*Hoxa9*-R: 5'-TCTCCGCCGCTCTCATTCTC-3'

Supplementary Figure 1. Uncropped gel of Figure 2C

Genomic PCR analysis of WT and *Gdf5*-HiBiT KI mice is shown. A boxed region indicates Figure 2C.

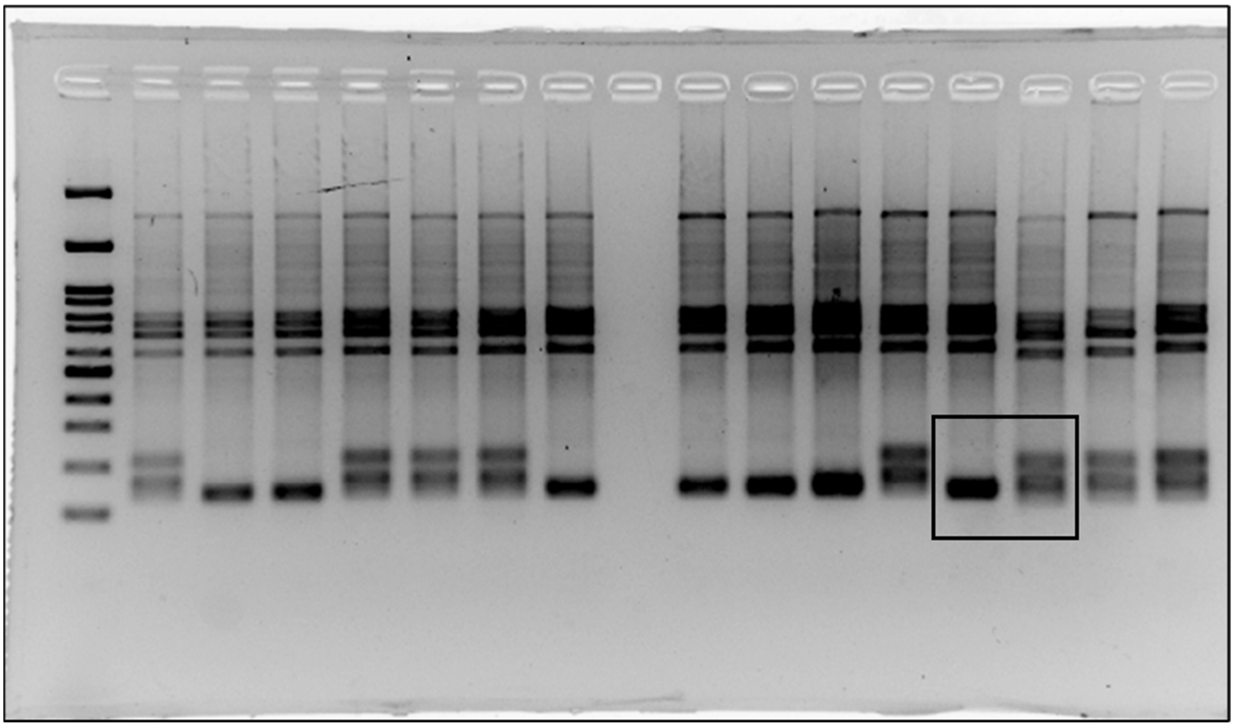

Supplement: Supplementary file 1 — Supplementary Information. [file 41598_2023_50318_MOESM1_ESM.pdf]
